# Ensemble of European regional climate simulations for the winter of 2013 and 2014 from HadAM3P-RM3P

**DOI:** 10.1038/sdata.2018.57

**Published:** 2018-04-10

**Authors:** Nathalie Schaller, Sarah N. Sparrow, Neil R. Massey, Andy Bowery, Jonathan Miller, Simon Wilson, David C.H. Wallom, Friederike E.L. Otto

**Affiliations:** 1 Environmental Change Institute, University of Oxford, Oxford OX1 3QY, UK; 2 Center for International Climate Research (CICERO), Gaustadalleen 21, 0349 Oslo, Norway; 3 Oxford e-Research Centre, University of Oxford, Oxford OX1 3QG, UK; 4 MetOffice Hadley Centre, Exeter EX1 3PB, UK

**Keywords:** Attribution, Environmental chemistry

## Abstract

Large data sets used to study the impact of anthropogenic climate change on the 2013/14 floods in the UK are provided. The data consist of perturbed initial conditions simulations using the Weather@Home regional climate modelling framework. Two different base conditions, Actual, including atmospheric conditions (anthropogenic greenhouse gases and human induced aerosols) as at present and Natural, with these forcings all removed are available. The data set is made up of 13 different ensembles (2 actual and 11 natural) with each having more than 7500 members. The data is available as NetCDF V3 files representing monthly data within the period of interest (1st Dec 2013 to 15th February 2014) for both a specified European region at a 50 km horizontal resolution and globally at N96 resolution. The data is stored within the UK Natural and Environmental Research Council Centre for Environmental Data Analysis repository.

## Background & Summary

In a warming world, it is increasingly important for decision-making and investments at the national and local scale to take into account changing patterns of weather and in particular extreme weather and climate-related events. While general information about climate change projections is widely available, decision-makers around the world are lacking information about how climate change may affect extreme weather events in their location. Having such information is particularly valuable in the aftermath of extreme and record breaking events, when politicians and policy officials are granted license to pursue more climate resilient policies and investments.

The widespread flooding in Southern England following the record breaking rainfall of January 2014 was such an event and is thus the subject of this study. The risk of flooding is determined not only by the hazard itself but also by the exposure and vulnerability and to address the true risk it is important to disentangle these different causes.

Focusing on precipitation during the winter 2013/2014 in Southern England we found that the likelihood of extreme precipitation similar to the observed event has increased by 0–160% depending on which of the naturalised Sea Surface Temperatures (SST) patterns, as described later, are used. In terms of information useful to decision-making, this means that while anthropogenic climate change does not decrease the likelihood of extreme precipitation, the change in frequency is also not so large that one could speak of a new normal. To successfully address future flood risk, a change in hazard is only a small part of the puzzle.

Data provided here allows the assessment of whether, and to what extent, anthropogenic climate change (and large scale internal climate drivers) altered the risk of extreme meteorological events. Whilst not directly addressing flooding *per se*, it allows analysis of the meteorological hazard over all of the European CORDEX-region^1^, providing a wide range of meteorological variables in an exceptionally large dataset, even by Weather@Home standards.

As shown previously^2^, datasets of this size are necessary to assess the likelihood of rare events as statistical fitting of extreme value distributions do not always result in the same behaviour of the distribution in the tails compared to large ensembles. The published dataset thus in particular allows to analyse rare events and can be used to test general assumptions made by applying extreme value theory to observed and modelled data and probabilistic event attribution studies^3^.

The primary analysis of this data^3^ provides presumably the first truly end-to-end extreme event attribution study (building on previous work^4^) by estimating how the increase in risk due to anthropogenic climate change propagates from an increase in the overall risk of extreme precipitation in Southern England in January and the associated changes in the atmospheric circulation to high river flows in the river Thames and inundated floodplains around Kingston. A subsequent publication^2^ demonstrates how changes in the atmospheric circulation depend on the observed SST in 2014 and hence highlight the necessity of defining a measure of the circulation processes causing the extreme event that is broad enough to not be beholden to the exact trajectory of the event but specific enough to represent the synoptic mechanisms. The methodology to separate changes in the atmospheric simulation from the overall change in risk^3^ has been developed further^5^ and alternative approaches proposed^6^.

All analyses undertaken with help of this data have focused so far on extreme precipitation in the UK, however the regional data covers the whole of Europe and recovers daily data for a number of variables including mean sea level pressure (mslp), wind speed and geopotential height rendering it an ideal tool to understand the drivers and atmospheric processes behind other unusual weather events of which an unusually high number occurred in the first quarter of 2014, globally and in Europe.

## Methods

Here, we describe both the method used to create the data initially, including the experimental framework and the method then available to subset and analyse the data once generated.

### Data Generation Method—The W@H simulation environment

**Figure 1:**
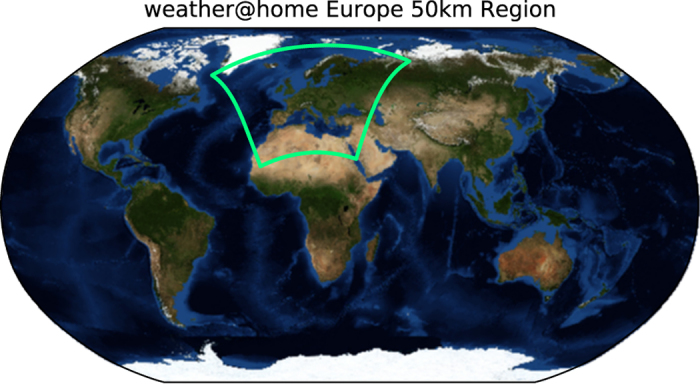
The region studied by the Regional Climate Model. Co-ordinates described in Table 1. The background image is the NASA ‘Blue Marble’ image available from http://visibleearth.nasa.gov.

The Weather@Home^7^ climate simulation environment uses the HadAM3P Atmosphere-only General Circulation Model (AGCM) with an embedded Regional Climate Model (RCM) variant, HadRM3P, both from the UK Met Office Hadley Centre. These models are based upon the atmospheric component of HadCM3 (ref. 8), a well documented and widely used coupled ocean—atmosphere model. HadRM3P is the regional model used in the Providing Regional Climates for Impacts Studies project (PRECIS)^9^, also originating from the UK Met Office. Within this dataset, the region studied by the RCM is defined as in Table 1 and shown in Fig. 1.

Both these models share the same model formulation, with differences in spatial resolution, timestep length and physical parameter values associated with length-scales. HadAM3P is integrated with a 15 min timestep, has 19 vertical levels and a horizontal resolution of 1.875^◦^ longitude and 1.25^◦^ latitude, which approximates to grid boxes of length ∼150 km at mid-latitudes and ∼200 km in the Tropics. HadRM3P also has 19 vertical levels, with a horizontal resolution of 50 km and a 5 min timestep. HadAM3P’s grid is defined as a regular latitude—longitude grid with regular poles, whereas HadRM3P employs a rotated grid, with artificial poles defined on a per-region basis so that the region of interest lies along the Equator in the rotated grid. Within a running instance the two models operate in an interleaved manner, alternating between the AGCM and RCM for each day of simulation time. This is a one way coupled configuration, i.e., at the end of each AGCM model day, the AGCM supplies boundary conditions to the RCM but there is no transfer in the other direction when the RCM has completed its model day.

**Figure 2:**
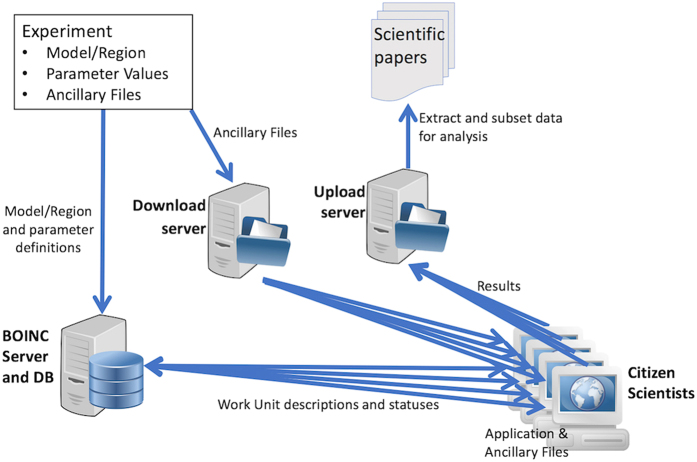
Workflow for submission of workunits within the Climateprediction.net/Weather@home system showing deposit of configuration and initial conditions data. The generated workunits distribution to citizen scientists systems, results passing back into the system and then onto the generation of scientific outputs.

An individual simulation is instantiated as a member of an experimental ensemble of which there will normally be a large number. The whole process is described in Fig. 2. Overall the experiment is one of many that are run within the Climateprediction.net(CPDN)^10^ program. CPDN uses the Berkeley Open Infrastructure for Network Computing (BOINC)^11^ framework as the mechanism for distribution of large number of individual computational tasks. This system utilizes the computational power of publically volunteered computers. Over its lifetime, CPDN has had over 630,000 computers registered of which approximately 20,000 may be considered recently active.

First the simulation application (AGCM and RCM) is copied to the download server, from where it is then distributed to all connected and active citizen scientists’ computers within the CPDN system. Then the project scientist must define the individual experiment. An experiment is made up of three components, the model and region definition, including the number of individual workunits in the ensemble, the models initial parameter values, definition of diagnostic outputs and ancillary files as used by both the AGCM and RCM. All inputs other than the experiment definition are copied to the download server for distribution to the citizen scientists’ computer. To ensure reproducibility we upload all experiment files to a repository so that when required a workunit or ensemble may be rerun. It is clear though that as the distribution of workunits onto citizen scientists client systems is random we cannot guarantee bi-wise reproducibility or an individually named workunit though we are able to guarantee statistical reproducibility^4^ and this is the basis of validation of the returned results.

The ensemble definitions are copied to the CPDN BOINC server which controls the distribution of the workunits to the volunteer systems. By submitting a large number of different ranges of inputs we allow the scientist to create ensembles of different related conditions and to understand the effects of these forcing changes. This also allows us to change the ‘type’ of world that a particular ensemble member is running. Within Weather@Home the most significant use of this is to either include or remove anthropogenically created greenhouse gases, sulphur dioxide and ozone from the atmosphere^12^ to perform an attribution experiment to assess the human impact on the chance of some form of extreme weather event occuring^13^. As each simulation instance is computed on the volunteer resources it returns its output to the projects upload server. Results are aggregated and presented to the project scientist as output data that can be further subsetted (using extraction scripts, https://github.com/CPDN-git/cpdn_extract_scripts) and analysed.

### Data Validation

During the development of the Weather@Home service there was significant work on the validation of the model outputs for the EU region^7^ used within the generation of the data described within this descriptor. During the running of a workunit on a volunteer system there are monthly returns of data, the presence and configuration of which are automatically checked by the BOINC client on the volunteers system. Loss of these before transmission, flags the workunit as failing, after which the BOINC system kills that workunit and relaunches it onto a different connected resource within the BOINC infrastructure. Only workunits that have returned a completed set of monthly uploads for the months of interest (DJFM) were used with incomplete workunits ignored and not uploaded. During the subsetting process the standard extraction scripts discard and results from workunits where corrupted data is detected.

### Code availability

The software models (HadAM3P and HadRM3) run to generate the data are open source and available from the UK Metoffice via the PRECIS website (http://www.metoffice.gov.uk/research/applied/applied-climate/precis). Alternatively the Climateprediction.net project simulation facility is open for collaboration from any group and has an academic license for the MetOffice software which can be shared with official collaborators.

## Data Records

The dataset contains the output from 13 different experiments as used in the initial analysis^3^. The details of these including experiment identifier, number of members in each ensemble, applied sea surface temperatures, atmospheric greenhouse gas concentrations and sea ice conditions initial conditions, are listed in Table 2.

In the Actual Conditions experiment, the AGCM uses observed SST data from 1 December 2013 until 15 February 2014 from the Operational Sea Surface Temperature and Sea Ice Analysis (OSTIA)^14^ dataset and present day atmospheric conditions (well-mixed greenhouse gases, ozone and reflective sulphate aerosols) to simulate weather events consistent with the observed climate. Due to the constraints of when simulations were run the last two weeks of February are driven with the average of the last available observed week (10–15 February 2014) as OSTIA data had yet to be released for those two weeks.

For the Natural experiments, estimates of the changes in SST patterns due to anthropogenic forcing based on 11 different coupled general circulation models (GCM) simulations from the Coupled Model Intercomparison Project phase 5(CMIP5)^15^ archive have been subtracted from the observed 2013/2014 SSTs used for the Actual Conditions simulations, and pre-industrial atmospheric composition is specified.

The Sea Ice input for the Actual experiment is OSTIA data for the study period where as for the Natural runs a Maximal extent of Sea Ice was constructed. Within this context Maximal extent when concerned with Sea Ice is the maximum observed extent of sea ice growth within the OSTIA record for both the northern and southern hemispheres.

The dataset as a whole is available from [Data Citation 1]. Subsets can be generated using the provided extraction scripts (https://github.com/CPDN-git/cpdn_extract_scripts). Each subset contains a number of individual workunit directories. These are a structured according to a template of different components as detailed below. First the workunit_name is defined as below;

*hadam3p_eu_<umid>_<start_year>_1_<workunit_id>*

e.g., hadam3p_eu_o72t_2013_1_008835758, of which the umid (o72t) is the important identifier denoting each ensemble member with other values either static within the dataset or of no real scientific value.

Since workunits may have failed when submitted to the CPDN infrastructure for a variety of independent reasons, CPDN configures its workunits to be regenerated up to 3 times if they suffer a failure, with previously returned results discarded. Therefore results are appended with a ‘regeneration’ number (either 00,1 or 22) giving a result directory name as:

*<workunit_name>_<0, 1 or 2>*

Therefore in the example, the directory is hadam3p_eu_o72t_2013_1_008835758_0.

Within each of these workunit directories there are four files as listed below;

- *<workunit_directory_filename>_1.zip* (Results from month 1, December)
- *<workunit_directory_filename>_2.zip* (Results from month 2, January)
- *<workunit_directory_filename>_3.zip* (Results from month 3, Febraury)
- *<workunit_directory_filename>_4.zip* (Results from month 4, March)

In the example we are using therefore the final filename is *hadam3p_eu_o72t_2013_1_008835758_0_1.zip*, i.e., a hadam3p workunit result file where UMID=o72t, the start year is 2013, the workunit ID is 008835758, it is a first generation workunit as regeneration number=0 and it contains the results for the first month of the run, December.

Within each result zip file are six files of which the three NetCDF formatted files as listed below are the scientific output.

- *<umid>ma.pc<year><month>.nc* file are the global model monthly mean
- *<umid>ga.pd<year><month>.nc* file is the regional model monthly mean
- *<umid>ga.pe<year><month>.nc* is the regional model daily average.

The year in this dataset is represented by either 13 or 14, 13 is 2013 and 14 2014.

Within each NetCDF file the reported diagnostics are returned as shown in Tables 3,4,5. Within these tables are the individual Field IDs of diagnostics, the standardized MetOffice code for these variables (the Stash Code^16^) the full name for the diagnostic according to the stash code and its units. The stash code, which identifies each of the individual diagnostics within the model and appears in the NetCDF file variable attributes for the output diagnostics (http://puma.nerc.ac.uk/STASH_to_CF/STASH_to_CF.html), is used for all analysis since the order within the NetCDF file of the output is not defined other than through the input configuration of the model. Therefore the Field ID number may not necessarily be constant across different utilisations of the model.

The header information of each file has been expanded within Supplementary Material using *ncdump –h*^17^, showing the declarations of dimensions, variables, and attributes of the example workunit results files.

## Technical Validation

**Figure 3:**
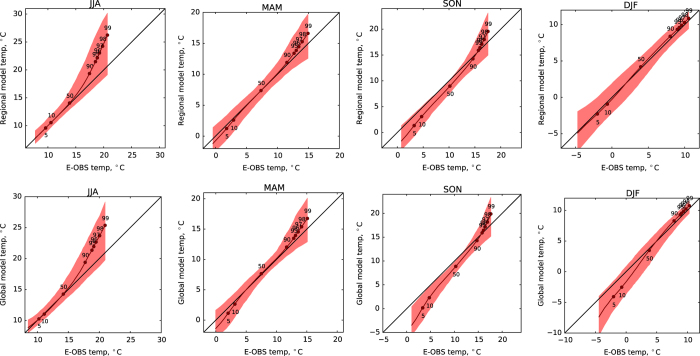
Quantile–quantile plot of the seasonal distribution of modeled daily mean temperature compared to the distribution of daily mean temperature in the E‐OBS dataset over the UK and Ireland. This is a reproduction of figure 12 (ref. 7). Columns from left to right represent MAM, JJA, SON and DJF respectively. The regional model is shown in the top row and the global model in the bottom row. The black line shows the quantile values for the entire ensemble. The red envelope shows the 5th to 95th percentile range of values for individual ensemble members.

**Figure 4:**
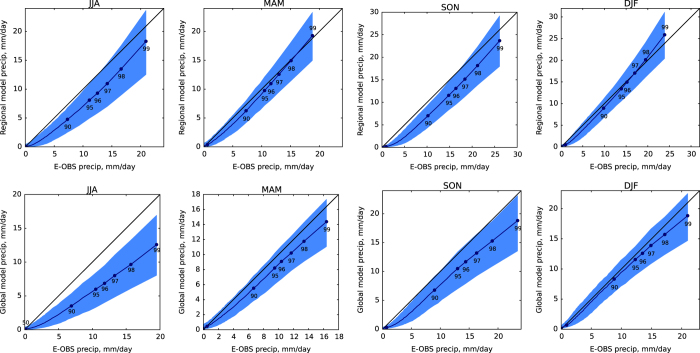
As figure 3 but for daily mean precipitation. This is a reproduction of figure 13 (ref. 7). The blue envelope shows the 5th to 95th percentile range of values for individual ensemble members.

The data published through this descriptor is a set of standard Weather@Home ensembles for the EU region. Therefore, for technical validation the mechanisms used within previous studies^7^ are valid here too. Of particular relevance are Figures 12 and 13 in that paper (here shown as Figs 3 and 4) where distributions of daily mean temperature and precipitation in both the global and regional models are compared with the E_OBS data over UK and Ireland.

For Schaller *et al.*^18^ the distribution of seasonal precipitation in Southern England was compared with observed data identifying a small wet bias. To analyse whether the model was able to simulate the main synoptic mechanisms behind the rainfall, simulated jet stream anomalies defined as zonal wind anomalies at 200 hPa were compared with reanalysis data (ERA-interim). The simulations showed good agreement as corroborated by a more detailed analysis on HadAM3P’s ability to simulate boreal winter weather^19^. This showed a good representation of the three modal jet steam behaviour compared to other state of the art general circulation models.

## Usage Notes

A set of scripts and software is available to utilise the data generated as a cohesive whole. These are available from https://github.com/CPDN-git/cpdn_extract_scripts, where you would use wah_extract_local.py to select the particular diagnostic set you wish to look at from across the dataset providing it with the location of the downloaded workunit files. A full set of documentation is available on how to use these scripts. This creates a cohesive set of output data which can then be viewed in any number of different tools of choice with NetCDF capabilities depending on user preference. The type of tool chosen will depend on the particular aim of the user with a variety of tools for interaction with NetCDF listed on a page maintained by the creators of the NetCDF standard, the Unidata program from UCAR (http://www.unidata.ucar.edu/software/netcdf/software.html).

## Additional information

**How to cite this article**: Schaller, N. *et al.* Ensemble of European regional climate simulations for the winter of 2013 and 2014 from HadAM3P-RM3P. *Sci. Data* 5:180057 doi: 10.1038/sdata.2018.57 (2018).

**Publisher’s note**: Springer Nature remains neutral with regard to jurisdictional claims in published maps and institutional affiliations.

## Supplementary Material



Supplementary Information

## Figures and Tables

**Table 1:** Corner co-ordinates for EU Region as used within dataset.

	**Longitude**	**Latitude**
Top left	53.7ºW	59.9ºN
Top right	76.5ºE	67.1ºN
Bottom right	38.5ºE	21.0ºN
Bottom left	11.5ºW	17.7ºN

**Table 2:** Sub experiments within the dataset with different Sea Surface Temperature.

**Experiment Identifier**	**Applied Sea Surface Temperatures**	**# of ensemble members**	**Atmospheric GHC conditions**	**Sea ice conditions**
a	2013/2014 OSTIA	17,367		2013/2014 OSTIA
c	2013/2014 OSTIA	9,067		Maximum extent[Northern hemisphere 1986/87, Southern Hemisphere 2007/08]
e	2013/2014 SSTs CanESM2 anthropogenic pattern	7243	Pre-industrial	
f	2013/2014 SSTs CCSM4 anthropogenic pattern	13989		
g	2013/2014 SSTs CNRM-CM5 anthropogenic pattern	7394		
h	2013/2014 SSTs CSIRO-Mk3-6-0 anthropogenic pattern	7595		
i	2013/2014 SSTs GFDL-CM3 anthropogenic pattern	15726		
j	2013/2014 SSTs GISS-E2-H anthropogenic pattern	15484		
k	2013/2014 SSTs GISS-E2-R anthropogenic pattern	7220		
l	2013/2014 SSTs HadGEM-ES anthropogenic pattern	11034		
m	2013/2014 SSTs IPSL-CM5A-LR anthropogenic pattern	7730		
n	2013/2014 SSTs IPSL-CM5A-MR anthropogenic pattern	10250		
o	2013/2014 SSTs MIROC-ESM anthropogenic pattern	13322		

**Table 3:** Diagnostic contents of Global Model Monthly results NetCDF file, *<umid>ma.pc<year><month>*.

**Field ID**	**Stash Code**	**Field Long Name as in file**	**Units**
1	16202	GEOPOTENTIAL HEIGHT: PRESSURE LEVELS	
8	16222	PRESSURE AT MEAN SEA LEVEL	Pa
16	3236	TEMPERATURE AT 1.5M	K
30	2204	Field30 (NET DOWN LW RAD FLUX: OPEN SEA)	W m^−2^
37	xx31	SEA ICE FRACTION AFTER TIMESTEP	
50	3249	10 METRE WIND SPEED M/S	m s^−1^
56	15201	U COMPNT OF WIND ON PRESSURE LEVELS	m s^−1^
57	15202	V COMPNT OF WIND ON PRESSURE LEVELS	m s^−1^
90	5216	TOTAL PRECIPITATION RATE KG/M2/S	kg m^−2^ s^−1^
93	xx23	SNOW AMOUNT AFTER TIMESTEP KG/M2	kg m^−2^
95	3227	Field 95 (SPECIFIC HUMIDITY AT 1.5M)	
106	8208	SOIL MOISTURE CONTENT	kg m^−2^
115	3229	Field115 (EVAP FROM SOIL SURF -AMOUNT KG/M2/TS)	kg m^−2^ s^−2^
180	3234	SURFACE LATENT HEAT FLUX W/M2	W m^−2^
186	1201	NET DOWN SURFACE SW FLUX: SW TS ONLY	W m^−2^
187	2201	NET DOWN SURFACE LW RAD FLUX	W m^−2^
200	1207	INCOMING SW RAD FLUX (TOA): ALL TSS	W m^−2^
201	1208	OUTGOING SW RAD FLUX (TOA)	W m^−2^
203	1235	TOTAL DOWNWARD SURFACE SW FLUX	W m^−2^
205	2207	DOWNWARD LW RAD FLUX: SURFACE	W m^−2^
206	2205	OUTGOING LW RAD FLUX (TOA)	W m^−2^
1530	8231	Field1530 (LAND SNOW MELT RATE KG/M2/S)	kg m^−2^ s^−1^
1532	8234	SURFACE RUNOFF RATE KG/M2/S	kg m^−2^ s^−1^
1533	8235	Field1533 (SUB-SURFACE RUNOFF RATE KG/M2/S)	kg m^−2^ s^−1^

**Table 4:** Diagnostic contents of Regional Model Monthly results NetCDF file, *<umid>ga.pe<year><month>*.

**Field ID**	**Stash Code**	**Field Long Name as within file**	**Units**
1	16202	GEOPOTENTIAL HEIGHT: PRESSURE LEVELS	m
8	16222	PRESSURE AT MEAN SEA LEVEL	Pa
16	3236	TEMPERATURE AT 1.5M	K
30	2204	field30 (NET DOWN LW RAD FLUX: OPEN SEA)	W m^−2^
37	000031	SEA ICE FRACTION AFTER TIMESTEP	
48	3225	10 METER WIND U-COMP	m s^−1^
49	3226	10 METER WIND V-COMP	m s^−1^
50	3249	10 METRE WIND SPEED M/S	m s^−1^
56	15201	U COMPNT OF WIND ON PRESSURE LEVELS	m s^−1^
57	15202	V COMPNT OF WIND ON PRESSURE LEVELS	m s^−1^
90	5216	TOTAL PRECIPITATION RATE KG/M2/S	kg m^−2^ s^−1^
93	000023	SNOW AMOUNT AFTER TIMESTEP KG/M2	kg m^−2^
95	3227	Field95 (SPECIFIC HUMIDITY AT 1.5M)	
106	8208	SOIL MOISTURE CONTENT	kg m^−2^
115	3229	Field115 (EVAP FROM SOIL SURF -AMOUNT KG/M2/TS)	kg m^−2^ s^−2^
180	3234	SURFACE LATENT HEAT FLUX W/M2	W m^−2^
186	1201	NET DOWN SURFACE SW FLUX: SW TS ONLY	W m^−2^
200	1207	INCOMING SW RAD FLUX (TOA): ALL TSS	W m^−2^
201	1208	OUTGOING SW RAD FLUX (TOA)	W m^−2^
203	1235	TOTAL DOWNWARD SURFACE SW FLUX	W m^−2^
205	2207	DOWNWARD LW RAD FLUX: SURFACE	W m^−2^
206	2205	OUTGOING LW RAD FLUX (TOA)	W m^−2^
1530	8231	Field1530 (LAND SNOW MELT RATE KG/M2/S)	kg m^−2^ s^−1^
1532	8234	SURFACE RUNOFF RATE KG/M2/S	kg m^−2^ s^−1^
1533	8235	Field1533 (SUB-SURFACE RUNOFF RATE KG/M2/S)	kg m^−2^ s^−1^

**Table 5:** Diagnostic contents of Regional Model Daily results NetCDF file, *<umid>ga.pd<year><month>*.

**Field ID**	**Stash Code**	**Field Long Name**	**Units**
1	16202	GEOPOTENTIAL HEIGHT: PRESSURE LEVELS	m
8	16222	PRESSURE AT MEAN SEA LEVEL	Pa
16	3236	TEMPERATURE AT 1.5M	K
50	3249	10 METRE WIND SPEED M/S	m s^−1^
88	3245	RELATIVE HUMIDITY AT 1.5M	
90	5216	TOTAL PRECIPITATION RATE KG/M2/S	kg m^−2^ s^−1^
186	1201	NET DOWN SURFACE SW FLUX: SW TS ONLY	W m^−2^
203	1235	TOTAL DOWNWARD SURFACE SW FLUX	W m^−2^
205	2207	DOWNWARD LW RAD FLUX: SURFACE	W m^−2^
