# Supplementary material for: Ensemble of European regional climate simulations for the winter of 2013 and 2014 from HadAM3P-RM3P
Source: Sci Data. 2018 Apr 10;5:180057. doi: 10.1038/sdata.2018.57 (PMC5892372; doi:10.1038/sdata.2018.57)
Supplement: Supplementary Information [file sdata201857-s2.docx]

# Supplementary Information

*Ensemble of EU Regional climate simulations for Winter 2013/14 from HadAM3P-RM3P*

Nathalie Schaller^1,2^, Sarah N. Sparrow^3^, Neil R. Massey^2^, Andy Bowery^3^, Jonathan Miller^3^, Simon Wilson^4^, David C.H. Wallom^3^ and Friederike E.L. Otto^2^

Description of results NetCDF files through expansion of the header information of each NetCDF file using ncdump –h, showing the declarations of dimensions, variables, and attributes of representative workunit results files.

o72tma.pcl3dec.nc (Global Model Monthly Averages)

netcdf o72tma.pcl3dec {

dimensions:

time0 = UNLIMITED ; // (1 currently)

time1 = 1 ;

z0 = 1 ;

z1 = 1 ;

z2 = 1 ;

z3 = 1 ;

z4 = 3 ;

z5 = 2 ;

z6 = 1 ;

z7 = 1 ;

latitude0 = 145 ;

longitude0 = 192 ;

latitude1 = 144 ;

longitude1 = 192 ;

variables:

float time0(time0) ;

time0:standard_name = "time" ;

time0:axis = "T" ;

time0:calendar = "360_day" ;

time0:units = "days since 2011-12-01 00:00:00" ;

time0:meaning_period = "720 hours" ;

time0:meaning_sampling_frequency = "3 hours" ;

float time1(time1) ;

time1:standard_name = "time" ;

time1:axis = "T" ;

time1:calendar = "360_day" ;

time1:units = "days since 2011-12-01 00:00:00" ;

time1:meaning_period = "720 hours" ;

time1:meaning_sampling_frequency = "1 hours" ;

float z0(z0) ;

z0:standard_name = "height" ;

z0:units = "m" ;

z0:direction = "up" ;

float z1(z1) ;

float z2(z2) ;

float z3(z3) ;

z3:standard_name = "height" ;

z3:units = "m" ;

z3:direction = "up" ;

float z4(z4) ;

z4:standard_name = "air_pressure" ;

z4:units = "hPa" ;

z4:direction = "down" ;

float z5(z5) ;

z5:standard_name = "air_pressure" ;

z5:units = "hPa" ;

z5:direction = "down" ;

float z6(z6) ;

z6:standard_name = "air_pressure" ;

z6:units = "hPa" ;

z6:direction = "down" ;

float z7(z7) ;

z7:standard_name = "height" ;

z7:units = "m" ;

z7:direction = "up" ;

float latitude0(latitude0) ;

latitude0:standard_name = "latitude" ;

latitude0:units = "degrees_north" ;

latitude0:axis = "Y" ;

float longitude0(longitude0) ;

longitude0:standard_name = "longitude" ;

longitude0:units = "degrees_east" ;

longitude0:axis = "X" ;

float latitude1(latitude1) ;

latitude1:standard_name = "latitude" ;

latitude1:units = "degrees_north" ;

latitude1:axis = "Y" ;

float longitude1(longitude1) ;

longitude1:standard_name = "longitude" ;

longitude1:units = "degrees_east" ;

longitude1:axis = "X" ;

float field186(time0, z0, latitude0, longitude0) ;

field186:_FillValue = -1.073742e+09f ;

field186:stash_item = "201" ;

field186:stash_section = "1" ;

field186:field_code = "186" ;

field186:standard_name = "surface_net_downward_shortwave_flux" ;

field186:long_name = "NET DOWN SURFACE SW FLUX: SW TS ONLY" ;

field186:units = "W m-2" ;

field186:cell_method = "time: mean " ;

float field200(time1, z1, latitude0, longitude0) ;

field200:_FillValue = -1.073742e+09f ;

field200:stash_item = "207" ;

field200:stash_section = "1" ;

field200:field_code = "200" ;

field200:standard_name = "toa_incoming_shortwave_flux" ;

field200:long_name = "INCOMING SW RAD FLUX (TOA): ALL TSS" ;

field200:units = "W m-2" ;

field200:cell_method = "time: mean " ;

float field201(time0, z1, latitude0, longitude0) ;

field201:_FillValue = -1.073742e+09f ;

field201:stash_item = "208" ;

field201:stash_section = "1" ;

field201:field_code = "201" ;

field201:standard_name = "toa_outgoing_shortwave_flux" ;

field201:long_name = "OUTGOING SW RAD FLUX (TOA)" ;

field201:units = "W m-2" ;

field201:cell_method = "time: mean " ;

float field203(time0, z0, latitude0, longitude0) ;

field203:_FillValue = -1.073742e+09f ;

field203:stash_item = "235" ;

field203:stash_section = "1" ;

field203:field_code = "203" ;

field203:standard_name = "surface_downwelling_shortwave_flux" ;

field203:long_name = "TOTAL DOWNWARD SURFACE SW FLUX" ;

field203:units = "W m-2" ;

field203:cell_method = "time: mean " ;

float field187(time0, z0, latitude0, longitude0) ;

field187:_FillValue = -1.073742e+09f ;

field187:stash_item = "201" ;

field187:stash_section = "2" ;

field187:field_code = "187" ;

field187:standard_name = "surface_net_downward_longwave_flux" ;

field187:long_name = "NET DOWN SURFACE LW RAD FLUX" ;

field187:units = "W m-2" ;

field187:cell_method = "time: mean " ;

float field30(time0, z2, latitude0, longitude0) ;

field30:_FillValue = -1.073742e+09f ;

field30:stash_item = 204 ;

field30:stash_section = 2 ;

field30:cell_method = "time: mean " ;

float field206(time0, z1, latitude0, longitude0) ;

field206:_FillValue = -1.073742e+09f ;

field206:stash_item = "205" ;

field206:stash_section = "2" ;

field206:field_code = "206" ;

field206:standard_name = "toa_net_upward_longwave_flux" ;

field206:long_name = "OUTGOING LW RAD FLUX (TOA)" ;

field206:units = "W m-2" ;

field206:cell_method = "time: mean " ;

float field205(time0, z0, latitude0, longitude0) ;

field205:_FillValue = -1.073742e+09f ;

field205:stash_item = "207" ;

field205:stash_section = "2" ;

field205:field_code = "205" ;

field205:standard_name = "surface_downwelling_longwave_flux" ;

field205:long_name = "DOWNWARD LW RAD FLUX: SURFACE" ;

field205:units = "W m-2" ;

field205:cell_method = "time: mean " ;

float field56(time1, z3, latitude1, longitude1) ;

field56:_FillValue = -1.073742e+09f ;

field56:stash_item = "225" ;

field56:stash_section = "3" ;

field56:field_code = "56" ;

field56:standard_name = "eastward_wind" ;

field56:long_name = "10 METRE WIND U-COMP" ;

field56:units = "m s-1" ;

field56:cell_method = "time: mean " ;

float field57(time1, z3, latitude1, longitude1) ;

field57:_FillValue = -1.073742e+09f ;

field57:stash_item = "226" ;

field57:stash_section = "3" ;

field57:field_code = "57" ;

field57:standard_name = "northward_wind" ;

field57:long_name = "10 METRE WIND V-COMP" ;

field57:units = "m s-1" ;

field57:cell_method = "time: mean " ;

float field115(time1, z0, latitude0, longitude0) ;

field115:_FillValue = -1.073742e+09f ;

field115:stash_item = 229 ;

field115:stash_section = 3 ;

field115:cell_method = "time: mean " ;

float field180(time1, z0, latitude0, longitude0) ;

field180:_FillValue = -1.073742e+09f ;

field180:stash_item = "234" ;

field180:stash_section = "3" ;

field180:field_code = "180" ;

field180:standard_name = "surface_upward_latent_heat_flux" ;

field180:long_name = "SURFACE LATENT HEAT FLUX W/M2" ;

field180:units = "W m-2" ;

field180:cell_method = "time: mean " ;

float field16(time1, z3, latitude0, longitude0) ;

field16:_FillValue = -1.073742e+09f ;

field16:stash_item = "236" ;

field16:stash_section = "3" ;

field16:field_code = "16" ;

field16:standard_name = "air_temperature" ;

field16:long_name = "TEMPERATURE AT 1.5M" ;

field16:units = "K" ;

field16:cell_method = "time: mean " ;

float field16_1(time1, z3, latitude0, longitude0) ;

field16_1:_FillValue = -1.073742e+09f ;

field16_1:stash_item = "236" ;

field16_1:stash_section = "3" ;

field16_1:field_code = "16" ;

field16_1:standard_name = "air_temperature" ;

field16_1:long_name = "TEMPERATURE AT 1.5M" ;

field16_1:units = "K" ;

field16_1:cell_method = "time: maximum " ;

float field16_2(time1, z3, latitude0, longitude0) ;

field16_2:_FillValue = -1.073742e+09f ;

field16_2:stash_item = "236" ;

field16_2:stash_section = "3" ;

field16_2:field_code = "16" ;

field16_2:standard_name = "air_temperature" ;

field16_2:long_name = "TEMPERATURE AT 1.5M" ;

field16_2:units = "K" ;

field16_2:cell_method = "time: minimum " ;

float field95(time1, z3, latitude0, longitude0) ;

field95:_FillValue = -1.073742e+09f ;

field95:stash_item = 237 ;

field95:stash_section = 3 ;

field95:cell_method = "time: mean " ;

float field50(time1, z3, latitude1, longitude1) ;

field50:_FillValue = -1.073742e+09f ;

field50:stash_item = "249" ;

field50:stash_section = "3" ;

field50:field_code = "50" ;

field50:standard_name = "wind_speed" ;

field50:long_name = "10 METRE WIND SPEED M/S" ;

field50:units = "m s-1" ;

field50:cell_method = "time: mean " ;

float field50_1(time1, z3, latitude1, longitude1) ;

field50_1:_FillValue = -1.073742e+09f ;

field50_1:stash_item = "249" ;

field50_1:stash_section = "3" ;

field50_1:field_code = "50" ;

field50_1:standard_name = "wind_speed" ;

field50_1:long_name = "10 METRE WIND SPEED M/S" ;

field50_1:units = "m s-1" ;

field50_1:cell_method = "time: maximum " ;

float field90(time1, z0, latitude0, longitude0) ;

field90:_FillValue = -1.073742e+09f ;

field90:stash_item = "216" ;

field90:stash_section = "5" ;

field90:field_code = "90" ;

field90:standard_name = "precipitation_flux" ;

field90:long_name = "TOTAL PRECIPITATION RATE KG/M2/S" ;

field90:units = "kg m-2 s-1" ;

field90:cell_method = "time: mean " ;

float field106(time1, z0, latitude0, longitude0) ;

field106:_FillValue = -1.073742e+09f ;

field106:stash_item = "208" ;

field106:stash_section = "8" ;

field106:field_code = "106" ;

field106:standard_name = "liquid_water_content_of_soil_layer" ;

field106:long_name = "SOIL MOISTURE CONTENT" ;

field106:units = "kg m-2" ;

field106:cell_method = "time: mean " ;

float field1530(time1, z0, latitude0, longitude0) ;

field1530:_FillValue = -1.073742e+09f ;

field1530:stash_item = 231 ;

field1530:stash_section = 8 ;

field1530:cell_method = "time: mean " ;

float field1532(time1, z0, latitude0, longitude0) ;

field1532:_FillValue = -1.073742e+09f ;

field1532:stash_item = "234" ;

field1532:stash_section = "8" ;

field1532:field_code = "1532" ;

field1532:standard_name = "surface_runoff_flux" ;

field1532:long_name = "SURFACE RUNOFF RATE KG/M2/S" ;

field1532:units = "kg m-2 s-1" ;

field1532:cell_method = "time: mean " ;

float field1533(time1, z0, latitude0, longitude0) ;

field1533:_FillValue = -1.073742e+09f ;

field1533:stash_item = 235 ;

field1533:stash_section = 8 ;

field1533:cell_method = "time: mean " ;

float field56_1(time1, z4, latitude1, longitude1) ;

field56_1:_FillValue = -1.073742e+09f ;

field56_1:stash_item = "201" ;

field56_1:stash_section = "15" ;

field56_1:field_code = "56" ;

field56_1:standard_name = "eastward_wind" ;

field56_1:long_name = "U COMPNT OF WIND ON PRESSURE LEVELS" ;

field56_1:units = "m s-1" ;

field56_1:cell_method = "time: mean " ;

float field57_1(time1, z5, latitude1, longitude1) ;

field57_1:_FillValue = -1.073742e+09f ;

field57_1:stash_item = "202" ;

field57_1:stash_section = "15" ;

field57_1:field_code = "57" ;

field57_1:standard_name = "northward_wind" ;

field57_1:long_name = "V COMPNT OF WIND ON PRESSURE LEVELS" ;

field57_1:units = "m s-1" ;

field57_1:cell_method = "time: mean " ;

float field95_1(time1, z6, latitude1, longitude1) ;

field95_1:_FillValue = -1.073742e+09f ;

field95_1:stash_item = 226 ;

field95_1:stash_section = 15 ;

field95_1:cell_method = "time: mean " ;

float field1(time1, z5, latitude0, longitude0) ;

field1:_FillValue = -1.073742e+09f ;

field1:stash_item = "202" ;

field1:stash_section = "16" ;

field1:field_code = "1" ;

field1:standard_name = "geopotential_height" ;

field1:long_name = "GEOPOTENTIAL HEIGHT: PRESSURE LEVELS" ;

field1:units = "m" ;

field1:cell_method = "time: mean " ;

float field16_3(time1, z4, latitude0, longitude0) ;

field16_3:_FillValue = -1.073742e+09f ;

field16_3:stash_item = "203" ;

field16_3:stash_section = "16" ;

field16_3:field_code = "16" ;

field16_3:standard_name = "air_temperature_at_pressure_level" ;

field16_3:long_name = "TEMPERATURE ON PRESSURE LEVELS" ;

field16_3:units = "K" ;

field16_3:cell_method = "time: mean " ;

float field8(time1, z7, latitude0, longitude0) ;

field8:_FillValue = -1.073742e+09f ;

field8:stash_item = "222" ;

field8:stash_section = "16" ;

field8:field_code = "8" ;

field8:standard_name = "air_pressure_at_sea_level" ;

field8:long_name = "PRESSURE AT MEAN SEA LEVEL" ;

field8:units = "Pa" ;

field8:cell_method = "time: mean " ;

float field93(time1, z0, latitude0, longitude0) ;

field93:_FillValue = -1.073742e+09f ;

field93:stash_item = "23" ;

field93:stash_section = "0" ;

field93:field_code = "93" ;

field93:standard_name = "surface_snow_amount" ;

field93:long_name = "SNOW AMOUNT AFTER TIMESTEP KG/M2" ;

field93:units = "kg m-2" ;

field93:cell_method = "time: mean " ;

float field37(time1, z0, latitude0, longitude0) ;

field37:_FillValue = -1.073742e+09f ;

field37:stash_item = "31" ;

field37:stash_section = "0" ;

field37:field_code = "37" ;

field37:standard_name = "sea_ice_area_fraction" ;

field37:long_name = "SEA ICE FRACTION AFTER TIMESTEP" ;

field37:units = "1" ;

field37:cell_method = "time: mean " ;

// global attributes:

:Conventions = "CF-1.3" ;

:alpham = 0.5f ;

}

o72tga.pdl3dec.nc (Regional Model Monthly Averages)

netcdf o72tga.pdl3dec {

dimensions:

time0 = UNLIMITED ; // (121 currently)

time1 = 120 ;

time2 = 30 ;

time3 = 30 ;

z0 = 1 ;

z1 = 1 ;

z2 = 1 ;

z3 = 1 ;

z4 = 1 ;

latitude0 = 119 ;

longitude0 = 122 ;

latitude1 = 118 ;

longitude1 = 122 ;

variables:

float time0(time0) ;

time0:standard_name = "time" ;

time0:axis = "T" ;

time0:calendar = "360_day" ;

time0:units = "days since 2011-12-01 00:00:00" ;

float time1(time1) ;

time1:standard_name = "time" ;

time1:axis = "T" ;

time1:calendar = "360_day" ;

time1:units = "days since 2011-12-01 00:00:00" ;

time1:meaning_period = "6 hours" ;

time1:meaning_sampling_frequency = "1 hours" ;

float time2(time2) ;

time2:standard_name = "time" ;

time2:axis = "T" ;

time2:calendar = "360_day" ;

time2:units = "days since 2011-12-01 00:00:00" ;

time2:meaning_period = "24 hours" ;

time2:meaning_sampling_frequency = "3 hours" ;

float time3(time3) ;

time3:standard_name = "time" ;

time3:axis = "T" ;

time3:calendar = "360_day" ;

time3:units = "days since 2011-12-01 00:00:00" ;

time3:meaning_period = "24 hours" ;

time3:meaning_sampling_frequency = "1 hours" ;

float z0(z0) ;

z0:standard_name = "air_pressure" ;

z0:units = "hPa" ;

z0:direction = "down" ;

float z1(z1) ;

z1:standard_name = "height" ;

z1:units = "m" ;

z1:direction = "up" ;

float z2(z2) ;

z2:standard_name = "height" ;

z2:units = "m" ;

z2:direction = "up" ;

float z3(z3) ;

z3:standard_name = "height" ;

z3:units = "m" ;

z3:direction = "up" ;

float z4(z4) ;

z4:standard_name = "air_pressure" ;

z4:units = "hPa" ;

z4:direction = "down" ;

float latitude0(latitude0) ;

latitude0:standard_name = "grid_latitude" ;

latitude0:units = "degrees" ;

latitude0:axis = "Y" ;

float longitude0(longitude0) ;

longitude0:standard_name = "grid_longitude" ;

longitude0:units = "degrees" ;

longitude0:axis = "X" ;

float global_latitude0(latitude0, longitude0) ;

global_latitude0:standard_name = "latitude" ;

global_latitude0:units = "degrees_north" ;

float global_longitude0(latitude0, longitude0) ;

global_longitude0:standard_name = "longitude" ;

global_longitude0:units = "degrees_east" ;

char rotated_pole0 ;

rotated_pole0:grid_mapping_name = "rotated_latitude_longitude" ;

rotated_pole0:grid_north_pole_latitude = 39.25f ;

rotated_pole0:grid_north_pole_longitude = 198.f ;

float latitude1(latitude1) ;

latitude1:standard_name = "grid_latitude" ;

latitude1:units = "degrees" ;

latitude1:axis = "Y" ;

float longitude1(longitude1) ;

longitude1:standard_name = "grid_longitude" ;

longitude1:units = "degrees" ;

longitude1:axis = "X" ;

float global_latitude1(latitude1, longitude1) ;

global_latitude1:standard_name = "latitude" ;

global_latitude1:units = "degrees_north" ;

float global_longitude1(latitude1, longitude1) ;

global_longitude1:standard_name = "longitude" ;

global_longitude1:units = "degrees_east" ;

char rotated_pole1 ;

rotated_pole1:grid_mapping_name = "rotated_latitude_longitude" ;

rotated_pole1:grid_north_pole_latitude = 39.25f ;

rotated_pole1:grid_north_pole_longitude = 198.f ;

float field1(time0, z0, latitude0, longitude0) ;

field1:_FillValue = -1.073742e+09f ;

field1:grid_mapping = "rotated_pole0" ;

field1:coordinates = "global_longitude0 global_latitude0" ;

field1:stash_item = "202" ;

field1:stash_section = "16" ;

field1:field_code = "1" ;

field1:standard_name = "geopotential_height" ;

field1:long_name = "GEOPOTENTIAL HEIGHT: PRESSURE LEVELS" ;

field1:units = "m" ;

float field8(time0, z1, latitude0, longitude0) ;

field8:_FillValue = -1.073742e+09f ;

field8:grid_mapping = "rotated_pole0" ;

field8:coordinates = "global_longitude0 global_latitude0" ;

field8:stash_item = "222" ;

field8:stash_section = "16" ;

field8:field_code = "8" ;

field8:standard_name = "air_pressure_at_sea_level" ;

field8:long_name = "PRESSURE AT MEAN SEA LEVEL" ;

field8:units = "Pa" ;

float field50(time1, z2, latitude1, longitude1) ;

field50:_FillValue = -1.073742e+09f ;

field50:grid_mapping = "rotated_pole1" ;

field50:coordinates = "global_longitude1 global_latitude1" ;

field50:stash_item = "249" ;

field50:stash_section = "3" ;

field50:field_code = "50" ;

field50:standard_name = "wind_speed" ;

field50:long_name = "10 METRE WIND SPEED M/S" ;

field50:units = "m s-1" ;

field50:cell_method = "time: maximum " ;

float field186(time2, z3, latitude0, longitude0) ;

field186:_FillValue = -1.073742e+09f ;

field186:grid_mapping = "rotated_pole0" ;

field186:coordinates = "global_longitude0 global_latitude0" ;

field186:stash_item = "201" ;

field186:stash_section = "1" ;

field186:field_code = "186" ;

field186:standard_name = "surface_net_downward_shortwave_flux" ;

field186:long_name = "NET DOWN SURFACE SW FLUX: SW TS ONLY" ;

field186:units = "W m-2" ;

field186:cell_method = "time: mean " ;

float field203(time2, z3, latitude0, longitude0) ;

field203:_FillValue = -1.073742e+09f ;

field203:grid_mapping = "rotated_pole0" ;

field203:coordinates = "global_longitude0 global_latitude0" ;

field203:stash_item = "235" ;

field203:stash_section = "1" ;

field203:field_code = "203" ;

field203:standard_name = "surface_downwelling_shortwave_flux" ;

field203:long_name = "TOTAL DOWNWARD SURFACE SW FLUX" ;

field203:units = "W m-2" ;

field203:cell_method = "time: mean " ;

float field205(time2, z3, latitude0, longitude0) ;

field205:_FillValue = -1.073742e+09f ;

field205:grid_mapping = "rotated_pole0" ;

field205:coordinates = "global_longitude0 global_latitude0" ;

field205:stash_item = "207" ;

field205:stash_section = "2" ;

field205:field_code = "205" ;

field205:standard_name = "surface_downwelling_longwave_flux" ;

field205:long_name = "DOWNWARD LW RAD FLUX: SURFACE" ;

field205:units = "W m-2" ;

field205:cell_method = "time: mean " ;

float field16(time3, z2, latitude0, longitude0) ;

field16:_FillValue = -1.073742e+09f ;

field16:grid_mapping = "rotated_pole0" ;

field16:coordinates = "global_longitude0 global_latitude0" ;

field16:stash_item = "236" ;

field16:stash_section = "3" ;

field16:field_code = "16" ;

field16:standard_name = "air_temperature" ;

field16:long_name = "TEMPERATURE AT 1.5M" ;

field16:units = "K" ;

field16:cell_method = "time: maximum " ;

float field16_1(time3, z2, latitude0, longitude0) ;

field16_1:_FillValue = -1.073742e+09f ;

field16_1:grid_mapping = "rotated_pole0" ;

field16_1:coordinates = "global_longitude0 global_latitude0" ;

field16_1:stash_item = "236" ;

field16_1:stash_section = "3" ;

field16_1:field_code = "16" ;

field16_1:standard_name = "air_temperature" ;

field16_1:long_name = "TEMPERATURE AT 1.5M" ;

field16_1:units = "K" ;

field16_1:cell_method = "time: minimum " ;

float field88(time3, z2, latitude0, longitude0) ;

field88:_FillValue = -1.073742e+09f ;

field88:grid_mapping = "rotated_pole0" ;

field88:coordinates = "global_longitude0 global_latitude0" ;

field88:stash_item = "245" ;

field88:stash_section = "3" ;

field88:field_code = "88" ;

field88:standard_name = "relative_humidity" ;

field88:long_name = "RELATIVE HUMIDITY AT 1.5M" ;

field88:units = "1" ;

field88:cell_method = "time: mean " ;

float field50_1(time3, z2, latitude1, longitude1) ;

field50_1:_FillValue = -1.073742e+09f ;

field50_1:grid_mapping = "rotated_pole1" ;

field50_1:coordinates = "global_longitude1 global_latitude1" ;

field50_1:stash_item = "249" ;

field50_1:stash_section = "3" ;

field50_1:field_code = "50" ;

field50_1:standard_name = "wind_speed" ;

field50_1:long_name = "10 METRE WIND SPEED M/S" ;

field50_1:units = "m s-1" ;

field50_1:cell_method = "time: maximum " ;

float field50_2(time3, z2, latitude1, longitude1) ;

field50_2:_FillValue = -1.073742e+09f ;

field50_2:grid_mapping = "rotated_pole1" ;

field50_2:coordinates = "global_longitude1 global_latitude1" ;

field50_2:stash_item = "249" ;

field50_2:stash_section = "3" ;

field50_2:field_code = "50" ;

field50_2:standard_name = "wind_speed" ;

field50_2:long_name = "10 METRE WIND SPEED M/S" ;

field50_2:units = "m s-1" ;

field50_2:cell_method = "time: mean " ;

float field90(time3, z3, latitude0, longitude0) ;

field90:_FillValue = -1.073742e+09f ;

field90:grid_mapping = "rotated_pole0" ;

field90:coordinates = "global_longitude0 global_latitude0" ;

field90:stash_item = "216" ;

field90:stash_section = "5" ;

field90:field_code = "90" ;

field90:standard_name = "precipitation_flux" ;

field90:long_name = "TOTAL PRECIPITATION RATE KG/M2/S" ;

field90:units = "kg m-2 s-1" ;

field90:cell_method = "time: mean " ;

float field1_1(time3, z4, latitude0, longitude0) ;

field1_1:_FillValue = -1.073742e+09f ;

field1_1:grid_mapping = "rotated_pole0" ;

field1_1:coordinates = "global_longitude0 global_latitude0" ;

field1_1:stash_item = "202" ;

field1_1:stash_section = "16" ;

field1_1:field_code = "1" ;

field1_1:standard_name = "geopotential_height" ;

field1_1:long_name = "GEOPOTENTIAL HEIGHT: PRESSURE LEVELS" ;

field1_1:units = "m" ;

field1_1:cell_method = "time: mean " ;

float field8_1(time3, z1, latitude0, longitude0) ;

field8_1:_FillValue = -1.073742e+09f ;

field8_1:grid_mapping = "rotated_pole0" ;

field8_1:coordinates = "global_longitude0 global_latitude0" ;

field8_1:stash_item = "222" ;

field8_1:stash_section = "16" ;

field8_1:field_code = "8" ;

field8_1:standard_name = "air_pressure_at_sea_level" ;

field8_1:long_name = "PRESSURE AT MEAN SEA LEVEL" ;

field8_1:units = "Pa" ;

field8_1:cell_method = "time: mean " ;

float field8_2(time3, z3, latitude0, longitude0) ;

field8_2:_FillValue = -1.073742e+09f ;

field8_2:grid_mapping = "rotated_pole0" ;

field8_2:coordinates = "global_longitude0 global_latitude0" ;

field8_2:stash_item = "1" ;

field8_2:stash_section = "0" ;

field8_2:field_code = "8" ;

field8_2:standard_name = "surface_air_pressure" ;

field8_2:long_name = "PSTAR AFTER TIMESTEP" ;

field8_2:units = "Pa" ;

field8_2:cell_method = "time: mean " ;

// global attributes:

:Conventions = "CF-1.3" ;

:alpham = 0.5f ;

}

o72tga.pel3dec.nc (Regional Model Daily Averages)

netcdf o72tga.pel3dec {

dimensions:

time0 = UNLIMITED ; // (1 currently)

time1 = 1 ;

z0 = 1 ;

z1 = 1 ;

z2 = 1 ;

z3 = 1 ;

z4 = 3 ;

z5 = 1 ;

z6 = 2 ;

z7 = 1 ;

latitude0 = 119 ;

longitude0 = 122 ;

latitude1 = 118 ;

longitude1 = 122 ;

variables:

float time0(time0) ;

time0:standard_name = "time" ;

time0:axis = "T" ;

time0:calendar = "360_day" ;

time0:units = "days since 2011-12-01 00:00:00" ;

time0:meaning_period = "720 hours" ;

time0:meaning_sampling_frequency = "3 hours" ;

float time1(time1) ;

time1:standard_name = "time" ;

time1:axis = "T" ;

time1:calendar = "360_day" ;

time1:units = "days since 2011-12-01 00:00:00" ;

time1:meaning_period = "720 hours" ;

time1:meaning_sampling_frequency = "1 hours" ;

float z0(z0) ;

z0:standard_name = "height" ;

z0:units = "m" ;

z0:direction = "up" ;

float z1(z1) ;

float z2(z2) ;

float z3(z3) ;

z3:standard_name = "height" ;

z3:units = "m" ;

z3:direction = "up" ;

float z4(z4) ;

z4:standard_name = "air_pressure" ;

z4:units = "hPa" ;

z4:direction = "down" ;

float z5(z5) ;

z5:standard_name = "air_pressure" ;

z5:units = "hPa" ;

z5:direction = "down" ;

float z6(z6) ;

z6:standard_name = "air_pressure" ;

z6:units = "hPa" ;

z6:direction = "down" ;

float z7(z7) ;

z7:standard_name = "height" ;

z7:units = "m" ;

z7:direction = "up" ;

float latitude0(latitude0) ;

latitude0:standard_name = "grid_latitude" ;

latitude0:units = "degrees" ;

latitude0:axis = "Y" ;

float longitude0(longitude0) ;

longitude0:standard_name = "grid_longitude" ;

longitude0:units = "degrees" ;

longitude0:axis = "X" ;

float global_latitude0(latitude0, longitude0) ;

global_latitude0:standard_name = "latitude" ;

global_latitude0:units = "degrees_north" ;

float global_longitude0(latitude0, longitude0) ;

global_longitude0:standard_name = "longitude" ;

global_longitude0:units = "degrees_east" ;

char rotated_pole0 ;

rotated_pole0:grid_mapping_name = "rotated_latitude_longitude" ;

rotated_pole0:grid_north_pole_latitude = 39.25f ;

rotated_pole0:grid_north_pole_longitude = 198.f ;

float latitude1(latitude1) ;

latitude1:standard_name = "grid_latitude" ;

latitude1:units = "degrees" ;

latitude1:axis = "Y" ;

float longitude1(longitude1) ;

longitude1:standard_name = "grid_longitude" ;

longitude1:units = "degrees" ;

longitude1:axis = "X" ;

float global_latitude1(latitude1, longitude1) ;

global_latitude1:standard_name = "latitude" ;

global_latitude1:units = "degrees_north" ;

float global_longitude1(latitude1, longitude1) ;

global_longitude1:standard_name = "longitude" ;

global_longitude1:units = "degrees_east" ;

char rotated_pole1 ;

rotated_pole1:grid_mapping_name = "rotated_latitude_longitude" ;

rotated_pole1:grid_north_pole_latitude = 39.25f ;

rotated_pole1:grid_north_pole_longitude = 198.f ;

float field186(time0, z0, latitude0, longitude0) ;

field186:_FillValue = -1.073742e+09f ;

field186:grid_mapping = "rotated_pole0" ;

field186:coordinates = "global_longitude0 global_latitude0" ;

field186:stash_item = "201" ;

field186:stash_section = "1" ;

field186:field_code = "186" ;

field186:standard_name = "surface_net_downward_shortwave_flux" ;

field186:long_name = "NET DOWN SURFACE SW FLUX: SW TS ONLY" ;

field186:units = "W m-2" ;

field186:cell_method = "time: mean " ;

float field200(time1, z1, latitude0, longitude0) ;

field200:_FillValue = -1.073742e+09f ;

field200:grid_mapping = "rotated_pole0" ;

field200:coordinates = "global_longitude0 global_latitude0" ;

field200:stash_item = "207" ;

field200:stash_section = "1" ;

field200:field_code = "200" ;

field200:standard_name = "toa_incoming_shortwave_flux" ;

field200:long_name = "INCOMING SW RAD FLUX (TOA): ALL TSS" ;

field200:units = "W m-2" ;

field200:cell_method = "time: mean " ;

float field201(time0, z1, latitude0, longitude0) ;

field201:_FillValue = -1.073742e+09f ;

field201:grid_mapping = "rotated_pole0" ;

field201:coordinates = "global_longitude0 global_latitude0" ;

field201:stash_item = "208" ;

field201:stash_section = "1" ;

field201:field_code = "201" ;

field201:standard_name = "toa_outgoing_shortwave_flux" ;

field201:long_name = "OUTGOING SW RAD FLUX (TOA)" ;

field201:units = "W m-2" ;

field201:cell_method = "time: mean " ;

float field203(time0, z0, latitude0, longitude0) ;

field203:_FillValue = -1.073742e+09f ;

field203:grid_mapping = "rotated_pole0" ;

field203:coordinates = "global_longitude0 global_latitude0" ;

field203:stash_item = "235" ;

field203:stash_section = "1" ;

field203:field_code = "203" ;

field203:standard_name = "surface_downwelling_shortwave_flux" ;

field203:long_name = "TOTAL DOWNWARD SURFACE SW FLUX" ;

field203:units = "W m-2" ;

field203:cell_method = "time: mean " ;

float field30(time0, z2, latitude0, longitude0) ;

field30:_FillValue = -1.073742e+09f ;

field30:grid_mapping = "rotated_pole0" ;

field30:coordinates = "global_longitude0 global_latitude0" ;

field30:stash_item = 204 ;

field30:stash_section = 2 ;

field30:cell_method = "time: mean " ;

float field206(time0, z1, latitude0, longitude0) ;

field206:_FillValue = -1.073742e+09f ;

field206:grid_mapping = "rotated_pole0" ;

field206:coordinates = "global_longitude0 global_latitude0" ;

field206:stash_item = "205" ;

field206:stash_section = "2" ;

field206:field_code = "206" ;

field206:standard_name = "toa_net_upward_longwave_flux" ;

field206:long_name = "OUTGOING LW RAD FLUX (TOA)" ;

field206:units = "W m-2" ;

field206:cell_method = "time: mean " ;

float field205(time0, z0, latitude0, longitude0) ;

field205:_FillValue = -1.073742e+09f ;

field205:grid_mapping = "rotated_pole0" ;

field205:coordinates = "global_longitude0 global_latitude0" ;

field205:stash_item = "207" ;

field205:stash_section = "2" ;

field205:field_code = "205" ;

field205:standard_name = "surface_downwelling_longwave_flux" ;

field205:long_name = "DOWNWARD LW RAD FLUX: SURFACE" ;

field205:units = "W m-2" ;

field205:cell_method = "time: mean " ;

float field48(time1, z3, latitude1, longitude1) ;

field48:_FillValue = -1.073742e+09f ;

field48:grid_mapping = "rotated_pole1" ;

field48:coordinates = "global_longitude1 global_latitude1" ;

field48:stash_item = "225" ;

field48:stash_section = "3" ;

field48:field_code = "56" ;

field48:standard_name = "eastward_wind" ;

field48:long_name = "10 METRE WIND U-COMP" ;

field48:units = "m s-1" ;

field48:cell_method = "time: mean " ;

float field49(time1, z3, latitude1, longitude1) ;

field49:_FillValue = -1.073742e+09f ;

field49:grid_mapping = "rotated_pole1" ;

field49:coordinates = "global_longitude1 global_latitude1" ;

field49:stash_item = "226" ;

field49:stash_section = "3" ;

field49:field_code = "57" ;

field49:standard_name = "northward_wind" ;

field49:long_name = "10 METRE WIND V-COMP" ;

field49:units = "m s-1" ;

field49:cell_method = "time: mean " ;

float field115(time1, z0, latitude0, longitude0) ;

field115:_FillValue = -1.073742e+09f ;

field115:grid_mapping = "rotated_pole0" ;

field115:coordinates = "global_longitude0 global_latitude0" ;

field115:stash_item = 229 ;

field115:stash_section = 3 ;

field115:cell_method = "time: mean " ;

float field180(time1, z0, latitude0, longitude0) ;

field180:_FillValue = -1.073742e+09f ;

field180:grid_mapping = "rotated_pole0" ;

field180:coordinates = "global_longitude0 global_latitude0" ;

field180:stash_item = "234" ;

field180:stash_section = "3" ;

field180:field_code = "180" ;

field180:standard_name = "surface_upward_latent_heat_flux" ;

field180:long_name = "SURFACE LATENT HEAT FLUX W/M2" ;

field180:units = "W m-2" ;

field180:cell_method = "time: mean " ;

float field16(time1, z3, latitude0, longitude0) ;

field16:_FillValue = -1.073742e+09f ;

field16:grid_mapping = "rotated_pole0" ;

field16:coordinates = "global_longitude0 global_latitude0" ;

field16:stash_item = "236" ;

field16:stash_section = "3" ;

field16:field_code = "16" ;

field16:standard_name = "air_temperature" ;

field16:long_name = "TEMPERATURE AT 1.5M" ;

field16:units = "K" ;

field16:cell_method = "time: mean " ;

float field16_1(time1, z3, latitude0, longitude0) ;

field16_1:_FillValue = -1.073742e+09f ;

field16_1:grid_mapping = "rotated_pole0" ;

field16_1:coordinates = "global_longitude0 global_latitude0" ;

field16_1:stash_item = "236" ;

field16_1:stash_section = "3" ;

field16_1:field_code = "16" ;

field16_1:standard_name = "air_temperature" ;

field16_1:long_name = "TEMPERATURE AT 1.5M" ;

field16_1:units = "K" ;

field16_1:cell_method = "time: maximum " ;

float field16_2(time1, z3, latitude0, longitude0) ;

field16_2:_FillValue = -1.073742e+09f ;

field16_2:grid_mapping = "rotated_pole0" ;

field16_2:coordinates = "global_longitude0 global_latitude0" ;

field16_2:stash_item = "236" ;

field16_2:stash_section = "3" ;

field16_2:field_code = "16" ;

field16_2:standard_name = "air_temperature" ;

field16_2:long_name = "TEMPERATURE AT 1.5M" ;

field16_2:units = "K" ;

field16_2:cell_method = "time: minimum " ;

float field95(time1, z3, latitude0, longitude0) ;

field95:_FillValue = -1.073742e+09f ;

field95:grid_mapping = "rotated_pole0" ;

field95:coordinates = "global_longitude0 global_latitude0" ;

field95:stash_item = 237 ;

field95:stash_section = 3 ;

field95:cell_method = "time: mean " ;

float field50(time1, z3, latitude1, longitude1) ;

field50:_FillValue = -1.073742e+09f ;

field50:grid_mapping = "rotated_pole1" ;

field50:coordinates = "global_longitude1 global_latitude1" ;

field50:stash_item = "249" ;

field50:stash_section = "3" ;

field50:field_code = "50" ;

field50:standard_name = "wind_speed" ;

field50:long_name = "10 METRE WIND SPEED M/S" ;

field50:units = "m s-1" ;

field50:cell_method = "time: mean " ;

float field50_1(time1, z3, latitude1, longitude1) ;

field50_1:_FillValue = -1.073742e+09f ;

field50_1:grid_mapping = "rotated_pole1" ;

field50_1:coordinates = "global_longitude1 global_latitude1" ;

field50_1:stash_item = "249" ;

field50_1:stash_section = "3" ;

field50_1:field_code = "50" ;

field50_1:standard_name = "wind_speed" ;

field50_1:long_name = "10 METRE WIND SPEED M/S" ;

field50_1:units = "m s-1" ;

field50_1:cell_method = "time: maximum " ;

float field90(time1, z0, latitude0, longitude0) ;

field90:_FillValue = -1.073742e+09f ;

field90:grid_mapping = "rotated_pole0" ;

field90:coordinates = "global_longitude0 global_latitude0" ;

field90:stash_item = "216" ;

field90:stash_section = "5" ;

field90:field_code = "90" ;

field90:standard_name = "precipitation_flux" ;

field90:long_name = "TOTAL PRECIPITATION RATE KG/M2/S" ;

field90:units = "kg m-2 s-1" ;

field90:cell_method = "time: mean " ;

float field106(time1, z0, latitude0, longitude0) ;

field106:_FillValue = -1.073742e+09f ;

field106:grid_mapping = "rotated_pole0" ;

field106:coordinates = "global_longitude0 global_latitude0" ;

field106:stash_item = "208" ;

field106:stash_section = "8" ;

field106:field_code = "106" ;

field106:standard_name = "liquid_water_content_of_soil_layer" ;

field106:long_name = "SOIL MOISTURE CONTENT" ;

field106:units = "kg m-2" ;

field106:cell_method = "time: mean " ;

float field1530(time1, z0, latitude0, longitude0) ;

field1530:_FillValue = -1.073742e+09f ;

field1530:grid_mapping = "rotated_pole0" ;

field1530:coordinates = "global_longitude0 global_latitude0" ;

field1530:stash_item = 231 ;

field1530:stash_section = 8 ;

field1530:cell_method = "time: mean " ;

float field1532(time1, z0, latitude0, longitude0) ;

field1532:_FillValue = -1.073742e+09f ;

field1532:grid_mapping = "rotated_pole0" ;

field1532:coordinates = "global_longitude0 global_latitude0" ;

field1532:stash_item = "234" ;

field1532:stash_section = "8" ;

field1532:field_code = "1532" ;

field1532:standard_name = "surface_runoff_flux" ;

field1532:long_name = "SURFACE RUNOFF RATE KG/M2/S" ;

field1532:units = "kg m-2 s-1" ;

field1532:cell_method = "time: mean " ;

float field1533(time1, z0, latitude0, longitude0) ;

field1533:_FillValue = -1.073742e+09f ;

field1533:grid_mapping = "rotated_pole0" ;

field1533:coordinates = "global_longitude0 global_latitude0" ;

field1533:stash_item = 235 ;

field1533:stash_section = 8 ;

field1533:cell_method = "time: mean " ;

float field56(time1, z4, latitude1, longitude1) ;

field56:_FillValue = -1.073742e+09f ;

field56:grid_mapping = "rotated_pole1" ;

field56:coordinates = "global_longitude1 global_latitude1" ;

field56:stash_item = "201" ;

field56:stash_section = "15" ;

field56:field_code = "56" ;

field56:standard_name = "eastward_wind" ;

field56:long_name = "U COMPNT OF WIND ON PRESSURE LEVELS" ;

field56:units = "m s-1" ;

field56:cell_method = "time: mean " ;

float field57(time1, z4, latitude1, longitude1) ;

field57:_FillValue = -1.073742e+09f ;

field57:grid_mapping = "rotated_pole1" ;

field57:coordinates = "global_longitude1 global_latitude1" ;

field57:stash_item = "202" ;

field57:stash_section = "15" ;

field57:field_code = "57" ;

field57:standard_name = "northward_wind" ;

field57:long_name = "V COMPNT OF WIND ON PRESSURE LEVELS" ;

field57:units = "m s-1" ;

field57:cell_method = "time: mean " ;

float field95_1(time1, z5, latitude1, longitude1) ;

field95_1:_FillValue = -1.073742e+09f ;

field95_1:grid_mapping = "rotated_pole1" ;

field95_1:coordinates = "global_longitude1 global_latitude1" ;

field95_1:stash_item = 226 ;

field95_1:stash_section = 15 ;

field95_1:cell_method = "time: mean " ;

float field1(time1, z6, latitude0, longitude0) ;

field1:_FillValue = -1.073742e+09f ;

field1:grid_mapping = "rotated_pole0" ;

field1:coordinates = "global_longitude0 global_latitude0" ;

field1:stash_item = "202" ;

field1:stash_section = "16" ;

field1:field_code = "1" ;

field1:standard_name = "geopotential_height" ;

field1:long_name = "GEOPOTENTIAL HEIGHT: PRESSURE LEVELS" ;

field1:units = "m" ;

field1:cell_method = "time: mean " ;

float field16_3(time1, z4, latitude0, longitude0) ;

field16_3:_FillValue = -1.073742e+09f ;

field16_3:grid_mapping = "rotated_pole0" ;

field16_3:coordinates = "global_longitude0 global_latitude0" ;

field16_3:stash_item = "203" ;

field16_3:stash_section = "16" ;

field16_3:field_code = "16" ;

field16_3:standard_name = "air_temperature_at_pressure_level" ;

field16_3:long_name = "TEMPERATURE ON PRESSURE LEVELS" ;

field16_3:units = "K" ;

field16_3:cell_method = "time: mean " ;

float field8(time1, z7, latitude0, longitude0) ;

field8:_FillValue = -1.073742e+09f ;

field8:grid_mapping = "rotated_pole0" ;

field8:coordinates = "global_longitude0 global_latitude0" ;

field8:stash_item = "222" ;

field8:stash_section = "16" ;

field8:field_code = "8" ;

field8:standard_name = "air_pressure_at_sea_level" ;

field8:long_name = "PRESSURE AT MEAN SEA LEVEL" ;

field8:units = "Pa" ;

field8:cell_method = "time: mean " ;

float field93(time1, z0, latitude0, longitude0) ;

field93:_FillValue = -1.073742e+09f ;

field93:grid_mapping = "rotated_pole0" ;

field93:coordinates = "global_longitude0 global_latitude0" ;

field93:stash_item = "23" ;

field93:stash_section = "0" ;

field93:field_code = "93" ;

field93:standard_name = "surface_snow_amount" ;

field93:long_name = "SNOW AMOUNT AFTER TIMESTEP KG/M2" ;

field93:units = "kg m-2" ;

field93:cell_method = "time: mean " ;

float field37(time1, z0, latitude0, longitude0) ;

field37:_FillValue = -1.073742e+09f ;

field37:grid_mapping = "rotated_pole0" ;

field37:coordinates = "global_longitude0 global_latitude0" ;

field37:stash_item = "31" ;

field37:stash_section = "0" ;

field37:field_code = "37" ;

field37:standard_name = "sea_ice_area_fraction" ;

field37:long_name = "SEA ICE FRACTION AFTER TIMESTEP" ;

field37:units = "1" ;

field37:cell_method = "time: mean " ;

// global attributes:

:Conventions = "CF-1.3" ;

:alpham = 0.5f ;

}
